# Cerebral Artery Pulsatility, Premorbid Blood Pressure, and Small Vessel Disease on Brain Imaging: A Population-Based Study

**DOI:** 10.1212/WNL.0000000000218181

**Published:** 2026-07-01

**Authors:** Linxin Li, Sara Mazzucco, Gary Kui Kai Lau, Maria Assuncao Tuna, Peter M. Rothwell

**Affiliations:** From the Wolfson Centre for Prevention of Stroke and Dementia, Nuffield Department of Clinical Neurosciences, Oxford University, United Kingdom.

## Abstract

**Background and Objectives:**

Increased pulsatility of cerebral blood flow is proposed as a potential cause of cerebral small vessel disease (SVD), but the apparent association could be confounded by shared vascular risk factors, especially current and past blood pressure (BP). Most previous studies were small, usually in selected populations, and most did not adjust fully for confounders, particularly heart rate, pulse pressure, and premorbid BP. Therefore, we aimed to determine if cerebral pulsatility is independently associated with SVD on brain imaging.

**Methods:**

Patients with TIA or minor ischemic stroke (NIH Stroke Scale ≤3) ascertained from 2011 to 2018 in a prospective population-based cohort study in Oxfordshire, the United Kingdom, were included. Cerebral pulsatility was measured with pulsatility index at the middle cerebral artery (MCA-PI) by transcranial Doppler ultrasound, and the burden of SVD was assessed using standard rating scales on MRI. We determined the associations of MCA-PI and SVD (total SVD score) adjusting for age, sex, vascular risk factors, heart rate, baseline BP (systolic, diastolic, and pulse pressure), and multiple premorbid BP readings up to 20 years before the event. We also performed mediation analysis to assess the contribution of MCA-PI on the causal pathway between BP and SVD.

**Results:**

Among 1,035 consecutive patients with measurements of both MCA-PI and SVD burden on MRI (mean age 66 years; 52.1% men), higher MCA-PI was associated with the total SVD score on MRI (top vs bottom quartile odds ratio [OR] 9.00, 95% CI 6.32–12.84). The association was attenuated after adjustment, but it remained significant (fully adjusted for systolic and diastolic BP OR 2.67, 1.55–4.60; for PP OR 1.88, 1.12–3.14). The results were consistent for individual markers of SVD (*p*-het = 0.77). In mediation analysis, 25.0% of the total association between premorbid systolic BP and SVD severity was due to MCA-PI.

**Discussion:**

In this cross-sectional analysis, cerebral pulsatility was positively associated with the increasing burden of cerebral SVD on brain MRI independent of age, sex, vascular risk factors, heart rate, baseline and premorbid BP in patients with TIA or minor ischemic stroke, and also explained a significant proportion of the association between BP and cerebral SVD on brain MRI. Prospective cohort studies and clinical trials targeting cerebral pulsatility are underway, which will help to further establish the role of cerebral pulsatility in SVD.

## Introduction

Cerebral small vessel disease (SVD) accounts for approximately 25% of all acute strokes, more than doubles the future risk of stroke and contributes to up to 45% of dementia.^1-3^ Increased pulsatility of cerebral blood flow has been proposed as a potential cause of SVD but evidence is conflicting. Some studies showed that cerebral pulsatility measured by the pulsatility index (PI) using transcranial Doppler ultrasound (TCD) was associated with the presence, severity, and progression of some markers of SVD such as lacunes and white matter hyperintensities (WMHs) in community volunteers,^4^ patients with hypertension,^5^ high-risk population,^6,7^ or selected patients with history of stroke.^8-10^ Other studies suggested that the apparent association of PI and SVD was mostly confounded by age and history of hypertension^11,12^ or was a much late manifestation as a result of SVD.^13^

However, most previous studies on PI at the middle cerebral artery (MCA-PI) were small (sample size 50–200) and did not adjust adequately for shared risk factors between MCA-PI and SVD. Importantly, no study adjusted for long-term premorbid mean blood pressure (BP), which should better reflect long-term exposure to increased BP better than history of hypertension,^14,15^ or compared the predictive value of PI with peripheral pulse pressure, which correlates with central pulsatility. Moreover, most studies focused on one marker of SVD rather than the overall burden.^4-10,16^ Furthermore, more studies that included nonselected patients with TIA or minor ischemic stroke, with the full range of manifestations and severities of SVD are needed to allow more detailed exploration of any “dose-effect” of the associations of PI and SVD.

We therefore aimed first to determine if cerebral pulsatility measured by MCA-PI on TCD was associated with the severity of the total burden of cerebral SVD measured on brain MRI independent of age, sex, vascular risk factors, heart rate, and baseline and multiple premorbid BP measurements in a cohort of unselected patients presenting with TIA or minor ischemic stroke. Second, we aimed to compare the predictive value of PI to the more easily measurable peripheral pulse pressure. Third, we assessed the contribution of MCA-PI on the causal pathway between BP and SVD and explored if the association of MCA-PI and SVD differed by patient characteristics.

## Methods

The Oxford Vascular Study (OXVASC) is an ongoing population-based study of the incidence and outcome of all acute vascular events.^17^ The study population comprises all 92,728 individuals, irrespective of age, registered with approximately 100 general practitioners (GPs) in 9 general practices in Oxfordshire, the United Kingdom.^17^ The multiple overlapping methods used to achieve near complete ascertainment of all individuals with TIA or ischemic stroke are detailed in the supplementary material (eMethods 1) and have been reported previously.^17,18^ This analysis includes consecutive adult patients (≥18 years) with a first-in-study TIA or minor ischemic stroke (NIH Stroke Scale ≤3) ascertained between June 1, 2011, and May 31, 2018.

In OXVASC, patients were seen by study physicians as soon as possible after the initial presentation. Baseline demographic data, premorbid use of preventive medication and premorbid vascular risk factors (i.e., hypertension, diabetes, hyperlipidemia, atrial fibrillation, smoking, history of coronary or peripheral vascular disease, history of TIA and history of stroke) were collected from face-to-face interview and cross-referenced with GP records.

Study nurses reviewed life-long patient records held in primary care and extracted all premorbid BP readings with dates up to 20 years before the event. We extracted data from both paper and computer records. Most readings were taken in the doctor's surgery by the physician or the practice nurse, for screening purposes, regular review, or an episode of minor illness. Measurements made during previous hospital admissions, often for major illness, were not recorded.^19^

At the first clinical assessment after TIA or ischemic stroke, as part of the OXVASC phenotyped cohort, routine TCD (Doppler box; Compumedics DWL, Singen, Germany) was performed with a 2 MHz hand-held probe by 2 trained neurologists following a standardized protocol (L.L. and S.M.). The 2 operators had excellent inter-rater reliability based on 50 scans (κ = 0.85) and were blinded to the results of the MRI scan. Two separate consecutive readings (including peak systolic velocity, end diastolic velocity, mean flow velocity, PI, and resistance index) are measured and recorded at the M1 segment (usually with depth 50–55 mm and at the depth with the optimal waveform when not adequate) of both the left and the right MCA, and a mean value was calculated for each marker. Systolic BP, diastolic BP, and end-tidal carbon dioxide levels were also measured immediately before and after the TCD measurements. Pulsatility is calculated using the Gosling equation: PI = (peak systolic velocity − end diastolic velocity)/mean velocity.^20^ PI characterizes the shape of the TCD waveform, and unlike peak systolic or diastolic velocity, PI is independent of the angle of the probe to the vessel of interest making it less operator-dependent.

All study patients were scanned predominantly with 2 scanners: Achieva (Philips Healthcare, Best, the Netherlands) and Magnetom Verio (Siemens Health Care, Munich, Germany). The detailed sequence parameters are listed in eTable 1.

MRI scans were assessed by 2 neurologists (L.L. and G.L.) for the total SVD score, with supervision from the study neuroradiologist.^21^ All assessors were blinded to the TCD measurements. As previously reported, one score is given for each of the SVD markers: (1) any cerebral microbleeds, which are rounded, hypodense foci up to 10 mm in size^22^; (2) any lacunes, which are defined as rounded or ovoid lesions between 3 and 20 mm in diameter, located in the basal ganglia, internal capsule, centrum semiovale or brainstem, that demonstrate CSF signal intensity on T2-weighted and fluid-attenuated inversion recovery sequences, without diffusion restriction; (3) enlarged perivascular spaces in the basal ganglia (more than 10), which are small (<3 mm) hyperintensities on T2 images; and (4) WMH (Fazekas scale for severe periventricular and/or moderate-to-severe deep WMH^23^). In addition, we also used the Age-Related White Matter Changes scale to determine the severity of WMH.^24^

### Statistical Analysis

We first compared the frequency of vascular risk factors, premorbid use of antihypertensive drugs, baseline as well as premorbid BP measurements, and MCA-PI between the 4 groups of the total SVD score using a χ^2^ test for *p*-trend for categorical variables and an ordinal regression for *p*-trend for continuous variables. In addition, *p* values adjusted for age and sex were also calculated to assess the age/sex-independent association of baseline characteristics and different SVD severity.

MCA-PI was then divided by quartiles, and the following variables were compared across the 4 groups: age, sex, prevalence of vascular risk factors and premorbid use of antihypertensive drugs, mean BP at assessment, and mean premorbid BP at different time points before the index event. Similarly, the χ^2^ test for *p*-trend for categorical variables and an ordinal regression for *p*-trend for continuous variables were calculated, and *p* values adjusted for age and sex were also assessed.

We used ordinal regression for the SVD score to determine the associations of MCA-PI and SVD burden in 5 different models: crude analysis; model 1: adjust for age and sex; model 2: model 1 with additional adjustment of vascular risk factors and premorbid use of antihypertensive drugs; model 3: model 2 with additional adjustment of baseline and premorbid BP; and model 4: model 3 plus adjustment for baseline and premorbid pulse pressure. Multicollinearity was tested in all adjusted models and the variance inflation factors were <2. Analyses were also stratified by age, sex, and history of hypertension. Given the association of pulsatility and heart rate,^25^ we also stratified the analysis by pulse rate and performed sensitivity analysis excluding those on a beta-blocker.

Premorbid BP measurements included most recent BP before the index event, mean BP <1, 1–5, 5–10 and >10 years prior, and long-term mean BP taking in all BP readings up to 20 years prior. We visually inspected the effect of the additional adjustment with each of these BP measurements on the common odds ratio (OR) for MCA-PI and the SVD score and the best measurement of the mean premorbid BP that has the most complete data were chosen for model 3.

For the mediation analysis, we used the *medeff* function from Stata and the SVD score was dichotomized into no-mild (SVD score ≤1) vs moderate-severe (SVD score ≥2) as only binary outcome is allowed in the model. All analyses were performed using Stata version 15.0.

### Standard Protocol Approvals, Registrations, and Patient Consents

Written informed consent or assent from relatives was obtained in all participants. OXVASC was approved by the local research ethics committee (OREC A: 05/Q1604/70).

### Data Availability

Requests for access to data should be submitted for consideration to the OXVASC Study Director (peter.rothwell@ndcn.ox.ac.uk).

## Results

Among 1,407 consecutive patients with suspected TIA or minor ischemic stroke, 1,313 (93.3%) had optimal temporal bone windows to record MCA-PI measured by TCD. Of the 1,313 who had measured MCA-PI, 1,055 (80.4%) were able to undergo brain MRI, of which 1,035 (98.1%) had adequate T2*, T2, or fluid-attenuated inversion recovery sequences performed for the visual rating of the SVD markers (eFigure 1). The common reasons for not having MRI included recent surgery, pacemaker, and not able to lie flat. Patients who did not have MRI were older (median/interquartile range [IQR] 71, 65–82 vs 69, 56–78, *p* < 0.0001), had higher mean MCA-PI (mean/SD 1.1/0.2 vs 0.97/0.2, *p* < 0.0001), and higher prevalence of diagnosed hypertension (65.0% vs 49.2%, *p* < 0.0001) than those that had MRI.

Among all 1,035 patients with measurements of both MCA-PI on TCD and SVD burden on MRI at the first assessment after the index event (median delay from symptom onset to assessment 4 days, IQR 2–12), 838 (81.0%) had the 2 investigations on the same day. All patients had at least 1 BP readings recorded before the stroke; 912 (88.1%; median number of readings 5, IQR 2–11), 965 (93.2%; median number 10, 3–22), and 833 (80.4%; median number 4, 1–12) had at least 1 reading within 5, 10, >10 years before the stroke, respectively.

The baseline characteristics of the 1,035 patients are presented in Table 1. In brief, the mean age was 66 years, and 52.1% were male. The commonest presenting event type was TIA (55.8%); 511 (49.4%) had history of hypertension, and 487 (47.1%) were on antihypertensive treatment before the index event. The mean/SD MCA-PI was 0.97/0.22. Using the total SVD score, 474 (45.8%) scored 0, 282 (27.2%) scored 1, 176 (17.0%) scored 2, and 103 (10.0%) scored ≥3.

**Table 1:** Baseline Characteristics of All Patients Included

	Included patients
Demographics	
Age (mean/SD)	66.0/14.8
Male sex	539 (52.1)
Risk factors	
Diagnosed hypertension	511 (49.4)
On antihypertensive drug	486 (47.0)
Calcium-channel blocker	199 (19.2)
ACEI/ARB	344 (33.2)
Beta-blocker	153 (14.8)
Diuretics	146 (14.1)
Other	77 (7.4)
Single agent	201 (19.4)
Multiple agents	285 (27.5)
Diabetes	118 (11.4)
Hyperlipidemia	334 (32.3)
Atrial fibrillation	97 (9.4)
Myocardial infarction	42 (4.1)
Peripheral vascular disease	34 (3.3)
Previous TIA	82 (7.9)
Previous stroke	85 (8.2)
Any history of smoking^a^	536 (51.9)
Current smoker^b^	138 (13.4)
SVD score (total SVD score)	
0	474 (45.8)
1	282 (27.2)
2	176 (17.0)
3–4	103 (10.0)
Baseline measures (mean/SD)	
Systolic blood pressure (mm Hg)	144/21.2
Diastolic blood pressure (mm Hg)	80.2/11.3
Heart rate	70.1/13.0
EtCO2	5.1/0.7
Transcranial Doppler measures of the middle cerebral artery (mean/SD)	
Peak systolic velocity (cm/s)	84.0/20.5
End diastolic velocity (cm/s)	34.2/10.8
Mean flow velocity (cm/s)	53.0/14.3
Pulsatility index	0.97/0.22
Resistance index	0.60/0.08

Abbreviations: ACEI = angiotensin-converting enzyme inhibitors; ARB = angiotensin II receptor blockers; EtCO2 = end-tidal carbon dioxide; SVD = small vessel disease.

Numbers are presented as number (%) unless otherwise specified.

a Number missing for 2.

b Number missing for 3.

Increasing SVD score was associated with higher MCA-PI but also with increasing age, higher prevalence of vascular risk factors including history of hypertension, premorbid use of antihypertensive medication, and high baseline as well as premorbid BP measurements (Table 2). Similarly, increasing MCA-PI was also strongly associated with age, history of hypertension, previous vascular disease, premorbid use of antihypertensive medication, and baseline as well as premorbid BP measurements (Table 3). Moreover, MCA-PI also correlated with present or premorbid pulse pressure (*r* = 0.50–0.64; eFigure 2).

**Table 2:** Associations of Demographics, Risk Factors, PI, and Blood Pressure Measurements With the Severity of Total SVD Burden

	SVD score				Age/sex-adjusted *p* value
	0 (n = 474)	1 (n = 282)	2 (n = 176)	3-4 (n = 103)	
Demographics					
Age (mean/SD)	58.0/14.7	69.6/11.1	75.2/10.0	77.5/10.2	—
Male sex	243 (51.3)	141 (50.0)	98 (55.7)	57 (55.3)	—
Risk factors					
Hypertension	161 (34.0)	154 (54.6)	120 (68.2)	76 (73.8)	<0.0001
Antihypertensives	151 (31.9)	147 (52.1)	119 (67.6)	70 (68.0)	<0.0001
Use of beta-blocker	51 (10.8)	43 (15.2)	38 (21.6)	21 (20.4)	0.73
Diabetes	43 (9.1)	41 (14.5)	18 (10.2)	16 (15.5)	0.49
Hyperlipidemia	117 (24.7)	98 (34.8)	79 (44.9)	40 (38.8)	0.15
Atrial fibrillation	19 (4.0)	33 (11.7)	28 (15.9)	17 (16.5)	0.005
Previous vascular disease	55 (11.6)	50 (17.7)	46 (26.1)	46 (44.7)	<0.0001
MI	14 (3.0)	8 (2.8)	13 (7.4)	7 (6.8)	0.31
PVD	8 (1.7)	12 (4.3)	3 (1.7)	11 (10.7)	0.10
Previous TIA	23 (4.9)	24 (8.5)	21 (11.9)	14 (13.6)	0.06
Previous stroke	21 (4.4)	16 (5.7)	26 (14.8)	22 (21.4)	<0.0001
History of smoking	233 (49.4)	159 (56.4)	91 (51.7)	53 (51.5)	0.31
MCA-PI (mean/SD)	0.88/0.18	0.98/0.20	1.08/0.23	1.14/0.24	<0.0001
BP (mm Hg) measurements (mean/SD)					
Baseline					
SBP	138.4/19.7	146.0/20.2	150.4/22.4	153.8/21.4	0.001
DBP	79.3/10.8	80.8/11.1	80.3/11.6	82.0/13.3	0.002
PP	59.1/16.3	65.2/17.1	70.0/18.7	71.9/17.4	0.08
MAP	99.0/12.1	102.6/12.3	104.1/13.5	106.4/14.3	0.0003
EtCO2	5.2/0.6	5.1/0.7	5.1/0.8	4.8/0.8	0.08
HR	69.8/13.0	70.3/13.2	70.6/13.4	70.3/11.5	0.22
Most recent premorbid	n = 447	n = 273	n = 171	n = 99	
SBP	130.3/15.4	133.8/14.8	135.4/14.8	139.5/16.4	0.08
DBP	76.5/10.2	75.9/9.2	75.3/8.1	76.1/10.7	0.19
PP	53.7/13.4	57.9/12.8	60.1/13.9	63.3/14.2	0.28
MAP	94.4/10.4	95.2/9.6	95.3/8.6	97.2/11.0	0.09
1 y premorbid	n = 284	n = 191	n = 126	n = 74	
SBP	132.5/14.8	137.4/12.8	136.1/12.3	140.6/13.2	0.10
DBP	76.9/9.9	77.1/8.3	75.9/7.9	76.8/8.5	0.03
PP	55.6/13.0	60.2/12.4	60.2/11.9	63.8/11.2	0.82
MAP	95.4/10.1	97.2/8.1	95.9/7.8	98.1/8.8	0.02
1–5 y premorbid	n = 373	n = 241	n = 153	n = 82	
SBP	131.3/14.1	136.3/12.7	138.0/11.3	139.6/12.3	0.03
DBP	77.7/9.1	77.9/7.6	76.7/6.9	76.9/7.7	0.08
PP	53.5/11.3	58.4/11.3	61.3/10.0	62.7/10.9	0.15
MAP	95.5/9.3	96.6/7.6	97.0/6.7	97.1/7.2	0.03
5–10 y premorbid	n = 359	n = 231	n = 146	n = 90	
SBP	130.2/14.3	136.4/12.6	138.8/11.3	141.8/13.1	<0.0001
DBP	79.0/8.6	79.3/7.2	78.9/7.8	79.8/8.8	0.10
PP	51.2/10.8	57.2/10.9	59.9/10.4	62.0/10.9	0.0002
MAP	96.1/9.6	98.3/7.8	98.9/7.6	100.4/9.1	0.003
>10 y premorbid	n = 373	n = 227	n = 146	n = 87	
SBP	128.3/15.2	134.4/14.9	139.2/15.4	141.2/13.6	0.005
DBP	77.5/9.2	80.7/7.7	81.8/8.2	81.9/7.3	0.02
PP	50.8/10.4	53.7/11.1	57.4/11.9	59.3/10.5	0.06
MAP	94.4/10.5	98.6/9.3	101.0/9.6	101.6/8.5	0.005
All available premorbid	n = 447	n = 273	n = 171	n = 99	
SBP	129.7/12.8	135.9/11.9	138.4/10.6	141.2/10.9	<0.0001
DBP	77.8/7.7	79.6/6.3	79.1/6.0	79.7/6.4	0.004
PP	51.9/9.4	56.3/10	59.3/9.2	61.5/9.7	0.001
MAP	95.2/8.6	98.4/7.2	99.0/6.4	100.5/6.8	0.0001

Abbreviations: BP = blood pressure; DBP = diastolic blood pressure; EtCO2 = end-tidal carbon dioxide; HR = heart rate; MAP = mean arterial pressure; MCA = middle cerebral artery; MI = myocardial infarction; PI = pulsatility index; PP = pulse pressure; PVD = peripheral vascular disease; SBP = systolic blood pressure; SVD = small vessel disease.

Numbers are presented as number (%) unless otherwise specified.

An ordinal regression is used to assess the age/sex adjusted differences between the 4 groups.

**Table 3:** Associations of Demographics, Risk Factors, and Blood Pressure Measurements With the Severity of Pulsatility Index Measured at the Middle Cerebral Artery on Transcranial Doppler

	MCA-PI quartiles				Age/sex-adjusted *p* value
	<0.81 (n = 257)	0.82–0.93 (n = 261)	0.94–1.09 (n = 257)	≥1.10 (n = 260)	
Demographics					
Age (mean/SD)	53.7/12.7	61.4/13.5	71.1/11.3	77.7/8.7	—
Male sex	135 (52.5)	147 (56.3)	126 (49.0)	131 (50.4)	—
Risk factors					
Hypertension	71 (27.6)	100 (38.3)	153 (59.5)	187 (71.9)	<0.0001
Antihypertensives	69 (26.8)	95 (36.4)	143 (55.6)	180 (69.2)	<0.0001
Use of beta-blocker	15 (5.8)	22 (8.4)	50 (19.5)	66 (25.4)	0.0002
Diabetes	11 (4.3)	25 (9.6)	32 (12.5)	50 (19.2)	<0.0001
Hyperlipidemia	49 (19.1)	81 (31.0)	91 (35.4)	113 (43.5)	0.02
Atrial fibrillation	17 (6.6)	19 (7.3)	26 (10.1)	35 (13.5)	0.25
Previous vascular disease	23 (8.9)	44 (16.9)	54 (21.0)	76 (29.2)	0.009
MI	2 (0.8)	11 (4.2)	11 (4.3)	18 (6.9)	0.06
PVD	1 (0.4)	5 (1.9)	8 (3.1)	20 (7.7)	0.002
Previous TIA	10 (3.9)	13 (5.0)	22 (8.6)	37 (14.2)	0.02
Previous stroke	15 (5.8)	21 (8.0)	25 (9.7)	24 (9.2)	0.69
History of smoking	125 (48.8)	148 (56.7)	129 (50.2)	134 (51.7)	0.28
BP (mm Hg) measurements (mean/SD)					
Baseline					
SBP	131.9/17.4	140.6/19.4	148.9/19.2	154.6/21.4	<0.0001
DBP	82.7/11.2	81.7/10.7	80.2/10.8	76.1/11.2	<0.0001
PP	49.2/10.9	59.0/12.5	68.7/13.7	78.5/17.8	<0.0001
MAP	99.1/12.6	101.3/13.0	103.1/12.6	102.3/12.9	0.87
HR	71.8/12.4	69.9/13.5	70.3/12.4	68.6/13.3	0.002
EtCO2	5.2/0.6	5.1/0.7	5.1/0.7	5.0/0.7	0.94
Most recent premorbid	n = 243	n = 245	n = 250	n = 252	
SBP	127.4/15.0	130.6/13.9	135.2/15.5	138.8/15.1	<0.0001
DBP	78.7/10.2	77.1/8.7	75.3/9.8	73.5/9.0	<0.0001
PP	48.7/10.9	53.5/11.0	59.9/12.9	65.3/14.0	<0.0001
MAP	94.9/10.9	94.9/9.4	95.3/10.4	95.2/9.3	0.46
1 y premorbid	n = 152	n = 162	n = 172	n = 189	
SBP	129.9/14.3	133.4/11.9	137.3/14.3	140.0/13.1	<0.0001
DBP	79.9/9.4	78.9/8.2	76.1/8.8	73.0/7.8	<0.0001
PP	50.0/10.0	54.5/8.9	61.1/11.6	67.0/12.5	<0.0001
MAP	96.6/10.2	97.1/8.6	96.5/9.5	95.3/7.9	0.08
1–5 y premorbid	n = 200	n = 204	n = 221	n = 224	
SBP	128.7/13.2	131.7/12.4	137.3/13.6	140.3/11.4	<0.0001
DBP	79.7/9.1	78.5/7.7	77.8/7.4	74.4/7.4	<0.0001
PP	48.9/8.6	53.1/8.9	59.6/10.5	65.9/10.2	<0.0001
MAP	96.0/9.8	96.2/8.6	97.6/8.6	96.4/7.6	0.90
5–10 y premorbid BP	n = 188	n = 196	n = 217	n = 225	
SBP	127.0/13.8	132.2/12.5	136.8/13.2	141.5/11.8	<0.0001
DBP	79.2/9.4	80.9/8.2	79.8/7.4	76.9/7.0	0.008
PP	47.8/9.0	51.3/8.5	57.0/10.5	64.5/10.1	<0.0001
MAP	95.1/10.2	98.0/9.0	98.8/8.4	98.4/7.5	0.16
>10 y premorbid BP	n = 181	n = 208	n = 218	n = 226	
SBP	123.8/12.9	128.9/14.1	136.0/15.1	142.1/14.3	<0.0001
DBP	76.5/9.4	79.4/8.9	81.0/8.6	80.8/7.1	0.48
PP	47.3/8.6	49.5/8.5	55.0/10.6	61.4/11.2	<0.0001
MAP	92.3/9.9	95.9/10.2	99.4/10.0	101.2/8.6	<0.0001
All premorbid	n = 243	n = 245	n = 250	n = 252	
SBP	126.8/11.5	131.0/11.3	136.7/12.1	141.5/10.9	<0.0001
DBP	78.8/7.7	79.3/7.0	79.3/6.7	77.5/6.2	0.004
PP	48.0/6.9	51.7/7.1	57.4/8.8	64.0/9.5	<0.0001
MAP	94.8/8.6	96.5/8.0	98.4/7.8	98.8/6.7	0.04

Abbreviations: BP = blood pressure; DBP = diastolic blood pressure; EtCO2 = end-tidal carbon dioxide; HR = heart rate; MAP = mean arterial pressure; MCA-PI = pulsatility index at the middle cerebral artery; PP = pulse pressure; PVD = peripheral vascular disease; SBP = systolic blood pressure.

Numbers are presented as number (%) unless otherwise specified.

In unadjusted analysis, the crude common OR was 9.00 (95% CI 6.32–12.84) for a shift in the total SVD score comparing patients in the top quartile of the MCA-PI with those in the bottom quartile. The OR attenuated to 1.91 (1.24–2.93) after adjustment for age and sex (Table 4), which was further attenuated with additional adjustment for vascular risk factors and use of antihypertensive medication (MCA-PI common OR 1.79, 1.15–2.79; Table 4).

**Table 4:** Associations of Cerebral Pulsatility Index and Small Vessel Disease Measured on Brain Imaging

	OR (95% CI)	*p* Value
Crude	9.00 (6.32–12.84)	<0.0001
Model 1. Age/sex adjusted	1.91 (1.24–2.93)	0.003
Model 2. Age/sex/risk factor adjusted	1.79 (1.15–2.79)	0.01
Model 3. Age/sex/risk factor/BP	2.67 (1.55–4.59)	<0.0001
Model 4. Age/sex/risk factor/PP	1.88 (1.12–3.14)	0.02

Abbreviations: BP = blood pressure; OR = odds ratio; PP = pulse pressure.

Model 1: adjusting for age and sex. Model 2: adjusting for age, sex, use of antihypertensive medication, history of hypertension, diabetes, hyperlipidemia, atrial fibrillation, history of myocardial infarction, peripheral vascular disease, previous TIA, or stroke. Model 3: model 2 plus systolic blood pressure and diastolic blood pressure at baseline as well as mean of all premorbid systolic and diastolic blood pressure. Model 4: model 2 plus pulse pressure at baseline and mean of all premorbid pulse pressure.

ORs representing the odds of increasing small vessel disease score in patients in the top quartiles of the middle cerebral artery pulsatility index vs patients in the bottom quartile (reference group).

We then compared the effect on the common ORs of MCA-PI and SVD with adjustment for different premorbid BP measurements (eFigure 3). The additional effect of premorbid mean BP on the common ORs was small and was broadly consistent when using mean BP measurements at different time points before the index event. We therefore included the long-term mean BP in the final model based on all available premorbid BP measurements.

In the fully adjusted model (Table 4), the association of MCA-PI and the SVD score remained significant (common OR 2.67, 1.55–4.60, *p* < 0.0001). The associations of MCA-PI and SVD were also present for individual markers of SVD (eFigure 4 and in fully adjusted model *p* for heterogeneity = 0.77) and was broadly consistent by stroke etiology (fully adjusted model − cardioembolic common OR 5.20, 1.09–24.82; small vessel 21.82, 2.63–181.12, cryptogenic 4.52, 1.63–12.51, large artery 1.87, 0.18–18.95; eTable 2). The results were also consistent in models adjusting for baseline and premorbid pulse pressure (Table 4) and in analyses where both pulse pressure and mean systolic BP were adjusted (eTable 3), with further adjustment for heart rate (eTable 3), or when excluding those on beta-blocker at baseline (eTable 3). In direct comparison with the predictive value with pulse pressure, MCA-PI was associated with SVD in all adjusted analyses, whereas the crude association between pulse pressure and SVD was attenuated and was no longer significant after adjustment. Moreover, in analyses where both MCA-PI and pulse pressure were included, there was no independent association between pulse pressure and SVD (eTable 4). Analyses were also stratified by age, sex, baseline pulse rate, and history of hypertension and showed no clear evidence of effect modification for age, sex, or pulse rate (Table 5). However, there was some evidence that the associations of MCA-PI and the severity of the total SVD burden were stronger in those without known history of hypertension or in those not on antihypertensive treatment.

**Table 5:** Associations of Cerebral Pulsatility Index and Total Small Vessel Disease Burden on Brain Imaging Stratified by Patient Age Groups, Sex, Diabetes, and History of Hypertension

	OR (95% CI)	OR (95% CI)	*p*-interaction
By age	<65 y (n = 426)	≥65 y (n = 609)	
Crude	3.15 (1.05–9.45)	3.10 (1.79–5.39)	0.77
Model 1	2.23 (0.72–6.94)	1.86 (1.03–3.33)	0.79
Model 2	2.59 (0.79–8.60)	1.73 (0.95–3.15)	0.92
Model 3	3.69 (1.02–13.34)	2.47 (1.23–4.99)	0.92
Model 4	2.79 (0.79–9.89)	1.78 (0.91–3.52)	0.90
By sex	Male (n = 539)	Female (n = 496)	
Crude	7.45 (4.61–12.05)	11.36 (6.70–19.27)	0.83
Model 1	2.28 (1.29–4.02)	1.40 (0.72–2.69)	0.70
Model 2	2.42 (1.34–4.37)	1.26 (0.63–2.51)	0.89
Model 3	3.14 (1.51–6.51)	2.28 (0.98–5.27)	0.91
Model 4	2.39 (1.18–4.80)	1.44 (0.65–3.16)	0.93
By history of hypertension	No (n = 524)	Yes (n = 511)	
Crude	11.05 (6.29–19.40)	5.48 (3.24–9.25)	0.05
Model 1	2.31 (1.19–4.48)	1.62 (0.88–3.01)	0.08
Model 2	2.48 (1.25–4.91)	1.76 (0.95–3.32)	0.08
Model 3	5.73 (2.49–13.19)	1.82 (0.85–3.91)	0.17
Model 4	3.48 (1.57–7.73)	1.52 (0.74–3.12)	0.05
By pulse rate	<70 (n = 535)	≥70 (n = 494)	
Crude	7.82 (4.74–12.89)	11.29 (6.75–18.87)	0.10
Model 1	2.06 (1.12–3.80)	2.09 (1.13–3.87)	0.28
Model 2	1.78 (0.94–3.37)	2.00 (1.05–3.81)	0.38
Model 3	2.75 (1.24–6.13)	2.48 (1.13–5.42)	0.61
Model 4	1.66 (0.80–3.47)	2.21 (1.03–4.75)	0.45
By use of antihypertensive medication at baseline	No (n = 549)	Yes (n = 486)	
Crude	15.13 (8.63–26.54)	3.76 (2.25–6.28)	<0.001
Model 1	3.46 (1.80–6.67)	1.15 (0.63–2.09)	0.02
Model 2	3.46 (1.77–6.74)	1.20 (0.64–2.25)	0.04
Model 3	6.18 (2.74–13.91)	1.32 (0.61–2.85)	0.07
Model 4	4.31 (1.97–9.43)	1.07 (0.52–2.20)	0.01

Abbreviation: OR = odds ratio.

ORs representing the odds of increasing small vessel disease score in patients in the top quartiles of the middle cerebral artery pulsatility index vs patients in the bottom quartile (reference group).

Model 1: adjusting for age and sex. Model 2: adjusting for age, sex, use of antihypertensive medication, history of hypertension, diabetes, hyperlipidemia, atrial fibrillation, history of myocardial infarction, peripheral vascular disease, previous TIA, or stroke. Model 3: model 3 plus systolic blood pressure and diastolic blood pressure at baseline as well as mean of all premorbid systolic and diastolic blood pressure. Model 4: model 3 plus pulse pressure at baseline and mean of all premorbid pulse pressure.

**Figure 1:**
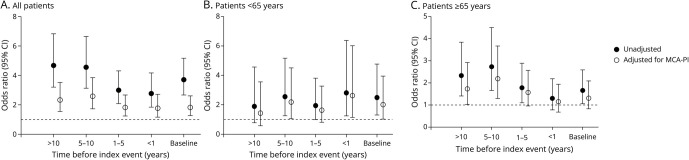
Modification Effect of Cerebral Pulsatility Index on the Associations of Premorbid Mean Systolic Blood Pressure and an Increasing Total Cerebral Small Vessel Disease Score MCA-PI = pulsatility index measured at the middle cerebral artery. Odds ratios of premorbid mean systolic blood pressure taken as top vs bottom quartile as the reference.

In crude mediation analysis (Figure 1), including MCA-PI attenuated the associations of premorbid mean systolic BP taken at any time point and the increasing total SVD score. The results were consistent in analysis stratified by age (Figure 1) or excluding those on beta-blockers (eFigure 5). After adjusting for age, sex, and other vascular risk factors, 15.2% (95% CI 9.5–37.4) and 25.0% (20.7–58.4) of the total effect between BP and SVD severity were due to MCA-PI for history of hypertension, and premorbid systolic BP, respectively.

## Discussion

In this population-based cohort of patients with TIA or minor ischemic stroke, we found that increasing MCA-PI was associated with increasing severity of the overall burden of cerebral SVD. This association was independent of age, sex, presence of vascular risk factors, heart rate, and baseline or premorbid levels of BP measurements, including both mean BP and pulse pressure. Moreover, MCA-PI also contributed to a significant proportion of the well-recognized association between BP and cerebral SVD.

The study results were in line with previous studies that showed positive associations between MCA-PI and WMH or lacunes in community volunteers, high-risk population, and in patients with lacunar stroke.^4-6,9,10^ The results were also consistent with 1 previous study that found strong association of increasing MCA-PI with moderate-to-severe WMH in patients with TIA or minor ischemic stroke.^8^ However, ours is the largest study to date to suggest that the observed association remains significant after adjustment for the key confounders (i.e., age, sex, vascular risk factors and different measurements of premorbid exposure to BP) and is consistent for individual components. Therefore, the apparent association between cerebral pulsatility and SVD was unlikely to be explained by shared common risk factors. This conclusion contradicts some other studies in the stroke-free population. Del Brutto et al.^11^ found in a group of 70 elderly volunteers that the adjusted mean MCA-PI was only numerically higher in those with more severe WMH (1.22 vs 1.10). Kneihsl et al.^12^ reported that the association of MCA-PI and WMH disappeared after adjustment for history of hypertension but with a very wide CI (adjusted OR 2.20, 95% CI 0.44–10.99).

Similar to all previous studies, given the cross-sectional design, the causal link between cerebral pulsatility and SVD cannot be assumed. However, emerging evidence suggests that MCA-PI is strongly dependent on aortic and MCA stiffness,^8,26-28^ indicating that increased MCA-PI representing stiffening large arteries could result in higher transmission of pulsatility to the cerebral small vessels causing endothelial dysfunction, altered cerebral autoregulation, low perfusion, and blood-brain-barrier disruption, ultimately leading to different manifestations of SVD.^7,8,27^ This could also partly explain why we found that MCA-PI mediated a significant proportion of the association between BP and SVD. Moreover, a treatment trial showed that drugs that decreased MCA-PI also tended to reduce the burden of WMH in patients with lacunar infarct, supporting MCA-PI as a cause of SVD.^29^ However, a recent cohort study found that baseline cerebral arterial PI measured on MRI was not associated with progression of WMHs, although they did not measure PI at follow-up.^30^ There were also other earlier studies suggesting that PI could reflect increased downstream resistance as a result of WMH.^13,31^ A longitudinal study measuring MCA-PI and SVD both at baseline and during follow-up is needed to understand if changes in SVD burden was associated with changes in MCA-PI.

Somewhat unexpectedly, in the multivariate analysis, we did not find that adjustment with long-term mean premorbid BP was significantly better than adjustment with history of hypertension alone perhaps partly due to the different effects of diastolic BP on SVD and on MCA-PI. Although higher diastolic BP was positively associated with the increasing SVD score, it was negatively associated with MCA-PI. The adjustment with premorbid use of antihypertensive drugs also did not appear better than the simple adjustment with history of hypertension, possibility due to variable compliance with medication and different potential effect of antihypertensive drug class on pulsatility.^32^ Moreover, although premorbid BP was strongly associated with arterial stiffness and current MCA-PI, a recent study have also showed that arterial stiffness was associated with SVD irrespective of concurrent BP in stroke-free individuals.^33^

There was some evidence suggesting that the association of MCA-PI and SVD might be stronger in those without known hypertension or in those not on antihypertensive treatment. Although the study was not powered to test the interaction reliably, the observation might be plausible as the association of MCA-PI and SVD could be explained by factors that were not related to BP.^13^ On the other hand, it is also possible that as increased MCA-PI and SVD become highly prevalent with increasing BP, TCD and visual rating of SVD score (with a range of 0–4) was not sensitive enough to differentiate between those above certain threshold. As a result of this “ceiling effect,” this study might have underestimated the association of MCA-PI and SVD in those with hypertension.

The strengths of the study include its population-based design, large sample size, comprehensive assessments of SVD markers, and detailed measurements of important confounders such as premorbid BP. However, there are limitations. First, only patients with recent TIA or minor ischemic stroke were included. Therefore, the study results cannot be generalized to more severe strokes, patients with previous strokes, or patients without strokes. However, given that over two-thirds of TIA or strokes are minor in nature^17,34^ and potential treatment trials in the area would also be likely to include stroke patients with less disability, the results may still be relevant in routine practice. Second, approximately 20% of the patients did not have an MRI scan mainly due to contraindications. Patients who did not have the MRI were older and had higher frequency of known hypertension and diabetes with presumably more severe cerebral SVD burden than those that completed the MRI assessment. They also had higher MCA-PI than those that had MRI scan. Therefore, excluding these patients could have caused underestimation of the association between MCA-PI and total SVD burden on imaging, although we still found the strong associations between the two. More data on the association of MCA-PI and SVD at older ages will also be helpful to understand if there is any interaction with age. Third, a small proportion of patients (6.7%) did not have adequate temporal bone windows, predominantly older women, for the assessment of MCA-PI using TCD. However, it is well-recognized that approximately 10% of the general population lack temporal bone windows and the association between MCA-PI and SVD was independent of age and sex. Fourth, a semiquantitative visual rating system was used to measure the burden of SVD on brain imaging, which might be less sensitive than quantitative measures. Although the visual rating approach is unlikely to be differential between the exposure groups as the assessment of SVD was blinded to the results of the MCA-PI measurements, it is still possible to dilute the true association between MCA-PI and cerebral SVD burden. We also did not include other potential markers for SVD, such as cerebral atrophy or cortical cerebral microinfarcts. Fifth, we used MCA-PI measured on TCD to assess cerebral pulsatility. MRI techniques are now also available with more direct and accurate measurement at the small vessel level, although perhaps much more costly.^10^ Sixth, some of our subgroup analyses may not be powered, for example, for those at younger ages. Moreover, although our analyses were hypothesis driven, we cannot rule out the effect of inflated type 1 error, particularly with multiple stratified analyses. Seven, our population is predominantly White and the results might not be fully generalizable to a more diverse population, who might have different susceptibility to increased pulsatility and SVD. Finally, we did not have data on other potential confounders such as BP variability and premorbid renal function.^35,36^ We also did not measure central arterial stiffness, which is an important determinant of cerebral pulsatility.^8^

Our results have both research and clinical implications. First, given that the most marked confounder for the associations of MCA-PI and SVD was age, future studies should always adjust for age. Second, we showed that the effect of adjustment for history of hypertension was largely comparable with the effect of adjusting for mean long-term premorbid BP suggesting that future studies without data on premorbid BP are unlikely to have major residual confounding when adjusting for baseline and history of hypertension alone. Third, although treating vascular risk factors is still vitally important for preventing cardiovascular disease in general, novel strategies targeting at other yet undetermined pathways might still be required to reduce the burden of cerebral SVD. Finally, if proved causal, treatment that directly reduces MCA-PI or systemic arterial stiffness might be a promising strategy for reducing cerebral pulsatlity and ultimately cerebral SVD burden.^29,31^

To conclude, we showed that cerebral pulsatility measured by MCA-PI was positively associated with increasing burden of cerebral SVD as measured on brain MRI in patients with TIA or minor ischemic stroke, independent of age, sex, vascular risk factors, heart rate, baseline, and premorbid BP measurements. Our findings suggest that the observed association of cerebral pulsatility and SVD burden was not fully explained by shared vascular risk factors. Future research with longitudinal data and measurement of aortic stiffness is required to confirm the causal link between cerebral pulsatility and SVD.

## Glossary

BP: blood pressure
GP: general practitioner
IQR: interquartile range
MCA-PI: PI at the middle cerebral artery
OR: odds ratio
OXVASC: Oxford Vascular Study
PI: pulsatility index
SVD: small vessel disease
TCD: transcranial Doppler ultrasound
WMH: white matter hyperintensity
